# Liver Cancer: Molecular Characterization, Clonal Evolution and Cancer Stem Cells

**DOI:** 10.3390/cancers9090127

**Published:** 2017-09-20

**Authors:** Germana Castelli, Elvira Pelosi, Ugo Testa

**Affiliations:** Department of Hematology, Oncology and Molecular Medicine, Istituto Superiore di Sanità, Rome 00141, Italy; germana.castelli@iss.it (G.C.); elvira.pelosi@iss.it (E.P.)

**Keywords:** hepatocellular carcinoma, cholangiocarcinoma, cirrhosis, hepatitis, hepatic adenoma, hepatic dysplastic nodules, cancer stem cells, tumor xenotrasplantation assay, gene sequencing

## Abstract

Liver cancer is the second most common cause of cancer-related death. The major forms of primary liver cancer are hepatocellular carcinoma (HCC) and intrahepatic cholangiocarcinoma (iCCA). Both these tumors develop against a background of cirrhotic liver, non-alcoholic fatty liver disease, chronic liver damage and fibrosis. HCC is a heterogeneous disease which usually develops within liver cirrhosis related to various etiologies: hepatitis B virus (HBV) infection (frequent in Asia and Africa), hepatitis C virus (HCV), chronic alcohol abuse, or metabolic syndrome (frequent in Western countries). In cirrhosis, hepatocarcinogenesis is a multi-step process where pre-cancerous dysplastic macronodules transform progressively into HCC. The patterns of genomic alterations observed in these tumors were recently identified and were instrumental for the identification of potential targeted therapies that could improve patient care. Liver cancer stem cells are a small subset of undifferentiated liver tumor cells, responsible for cancer initiation, metastasis, relapse and chemoresistance, enriched and isolated according to immunophenotypic and functional properties: cell surface proteins (CD133, CD90, CD44, EpCAM, OV-6, CD13, CD24, DLK1, α2δ1, ICAM-1 and CD47); the functional markers corresponding to side population, high aldehyde dehydrogenase (ALDH) activity and autofluorescence. The identification and definition of liver cancer stem cells requires both immunophenotypic and functional properties.

## 1. Introduction

Liver cancer is one of the major cause of cancer-related death in the world; in fact, this malignancy is the second most common cause of cancer-related death and its incidence and mortality rates are increasing [1,2,3]. It was estimated that about 700,000 deaths worldwide annually are related to this cancer. In the United States liver cancer is the neoplasm with the greatest increase in mortality during the past two decades. Primary liver cancers comprise a group of aggressive tumors, characterized by a consistent ethnic, etiological and geographical diversity. The major forms of primary liver cancer are hepatocellular carcinoma (HCC) and intrahepatic cholangiocarcinoma (iCCA). Both these tumors develop against a background of cirrhotic liver, non-alcoholic fatty liver disease and chronic liver damage and fibrosis. In addition, there are two rare malignant liver tumors occurring in adolescence—hepatoblastoma and fibrolamellar carcinoma—both developing in a non-cirrhotic liver. HCC is one of the cancers whose incidence and mortality increase in the world at the fastest rate, is more frequent in males (male/female ratio of about 3–5/1) and in Africa and Asia than in Europe. The major risk factors for this cancer are represented by infection with hepatitis B virus, chronic hepatitis C virus infection, alcoholic liver disease and non-alcoholic fatty liver disease and metabolic disorders, such as hemochromatosis [1,2,3]. iCCA is the second most common form of primary liver cancer, whose incidence and worldwide mortality are also increasing. This tumor also displays consistent racial and ethnic differences, with the highest incidence observed in Hispanic and Asian ethnicities. Risk factors for development of iCCA include primary sclerosing cholangitis, biliary duct cystis, hepatolithiasis and parasitic biliary infestations with flukes. Furthermore, shared risk factors with HCC represented by HCV and HBV [1,2,3].

Although HCC and iCCA are related to different etiological agents, both these primary liver cancers are strictly linked in their development to chronic liver damage, whereby a chronic liver disease causes a constant liver cell damage, resulting in a high cell turnover, a condition favoring the occurrence of errors related to the chronic repair processes. Because of these chronic reparation processes, the hepatic microenvironment is markedly altered, resulting in the generation of an oncogenic microenvironment, creating a pro-oncogenic milieu and thus promoting liver cell transformation.

Curative therapies are possible only for early-stage tumors and consist in resection, local ablation or liver transplantation [2]. At more advanced stages, no curative treatments are available and only chemoembolization or treatment with the multikinase inhibitor sorafenib (Nexavar^®^) have shown some benefits in terms of patient survival [2].

Cellular and molecular studies have shown that adult hepatocytes are the cells of origin ol liver tumors, with different possible pathways of hepatocarcinogenesis: these cells directly transform into HCC through the progressive acquisition of specific genetic alterations or de-differentiate into hepatocyte precursor cells which transform to HCC following the acquisition of genetic alterations or transdifferentiate into cells of the biliary lineage, whose malignant transformation generates iCCA [3].

In this review, we summarize the current understanding of the biology of liver cancers, moving from the study of genetic alterations, to the analysis of tumor heterogeneity and clonal evolution, and, finally, to the studies on the identification and characterization of cells initiating and maintaining these tumors.

## 2. Genetic Abnormalities in Liver Cancer

Numerous somatic genetic alterations have been observed at the level of HCC, including mutations, genes copy number alterations and intra-chromosomal and inter-chromosomal rearrangements. These studies have shown that the genomic landscape of primary liver cancers is highly complex, its complexity being further amplified by an association of these tumors with various etiological factors that precede tumor development by many years. Frequent gene alterations were observed at the level of various genes playing a key role in cancer development, such as TP53, MYC, WNT, CTNNB1 (beta-catenin), cell-cycle related genes CCND1 (cyclin D1) and CDKN2A (cyclin-dependent kinase inhibitor 2A); furthermore, are frequently altered genes involved in the control of telomere stability (telomerase reverse transcriptase, TERT), in epigenetic mechanisms (IDH1 and IDH2) and chromatin remodeling (ARID1, ARID2, MLL, BAP1 and EZH2) [4,5]. Importantly, 11 pathways are frequently altered in HCCs: TERT promoter mutations activating telomerase expression (about 60%), WNT/beta-catenin (54%), PI3K-AKT-mTOR (51%), TP53/cell cycle (49%), MAPK (43%), hepatic differentiation (34%), epigenetic regulation (32%), chromatin remodeling (28%), oxidative stress (12%), IL-6/JAK-STAT (9%) and TGF-beta [6]. Interestingly, genetic alterations in CTNNB1 and AXIN1, although belonging to the same pathway, are mutually exclusive; similarly, inactivating mutations of TP53 are rarely observed in association with CTNNB1 and AXIN1 mutations. TP53 and BAP1 mutations are also almost exclusive. A significant correlation between mutations and risk factors was observed: thus, alcohol-related HCCs were clearly enriched in CTNNB1, TERT, CDKN2A, SMARCA2 and HGF alterations, while HBV-related HCCs are enriched in reactivating TP53 mutations and KTM2B, leading to a more frequent involvement of cell cycle control [6]. Importantly, 28% of HCC patients harbored at least one damaging alteration targetable by an approved drug and about 86% of them harbored a mutation targetable by a drug studied in phase I to phase III clinical trials [6]. Totoki and coworkers have recently reported a screening on a very large number of liver cancer genomes (503) allowing to identify 30 candidate driver genes and 11 core pathways [7]. This study stratified the HCC patients according to the main etiological factors associated with this tumor type: HBV-positive; HCV-positive; non-HBV, non-HCV positive. TERT gene abnormalities were very frequently observed in all these three HCC subtypes, implicating TERT as a central ancestry-independent node in the pathogenic development of liver cancer [7]. Interestingly, multiple types of TERT genetic alterations are observed in HCC: a first type is represented by TERT promoter mutations, observed in 64% of HCV-related HCCs, in 59% of non-viral cases and 37% of HBV-positive cases; a second type is represented by TERT amplification observed in about 7% of cases; a third type is related to insertion of the HBV genome in the TERT locus, observed in 22% of HBV-positive HCCs [7]. Interestingly, TERT promoter mutations significantly co-occurred with WNT pathway gene alterations [7]. These authors observed also that some oncogenic pathways are activated in HCCs. Alterations of components of the TP53-RB pathway were observed in 72% of HCCs, including TP53 mutation (31% of cases), RB1 mutations (about 5% of cases), CDKN1A (frequently mutated), CDKN2A gene encoding p16 (frequent focal deletions), the two genes ATM and RPS6KA3 of the TP53 pathway (frequently mutated). Alterations of WNT pathway are observed in 66% of HCCs, including activating mutations of CTNNB1 mutations and inactivating mutations of AXIN1 and APC [7]. Alterations of chromatin and transcriptional modulators are frequently observed in HCCs: notable examples are the frequent alterations in NFE2L2, a transcriptional regulator activating antioxidant and cytoprotective genes, mutations of the nucleosome remodelers ARID1A, ARID2 and BRD7, copy number alterations of six members of the SWI/SNF complex [7]. Alterations of mTOR-PIK3CA pathway are observed in 45% of HCCs and are mainly represented by inactivating mutations in TSC1-TSC2, activating mutations and copy gain in PIK3CA, PTEN deletions, STK11 mutations [7]. The study of more than 600 HCC patients confirmed that TERT promoter mutations were the most common somatic mutation observed in these tumors, with a preferential occurrence in older patients, predominantly male, HCV positive; interestingly, TERT promoter-mutated HCCs displayed a strong co-occurrence of CDKN2A silencing by promoter hypermethylation [8].

**Figure 1:**
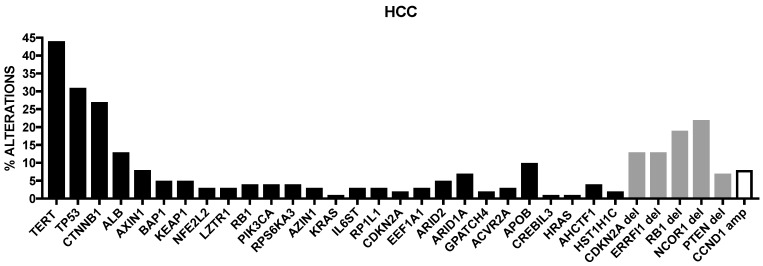
Main genetic alterations (gene mutations, gene deletions and amplifications) observed in HCC patients of multiethnic origin. The data are reprinted from a recent study of the cancer Genome Atlas Network [5] and are derived from a study based on the analysis of 363 patients.

Cancer Genome Atlas Research has recently published the results of a comprehensive and integrative genomic characterization of hepatocellular carcinoma [8] (Figure 1). At the level of somatic mutations, 26 genes were found to be frequently mutated. Most of these genes were previously identified in previous reports [4,5,6,7]. Among the already known mutations, Albumin (ALB) and APOB mutations were observed in 13% and 10% of tumors, in line with a previous report [9]. Driver mutational events were observed also at the level of LZTR1, EEF1A1, SMARCA4, AZIN1, RP1L1, GPATCH4, CREB3L3, AHCTF1 and HIST1H1c genes [8]. In this important study, it was also analyzed the methylation profiling of HCCs, providing evidence about the existence of four methylation clusters: the cluster 1 was characterized by relatively frequent TP53 mutations, virtually absent TERT promoter mutation, rare CDKN2A silencing, frequent HBV and rare HCV and frequent Asian ethnicity; the cluster 2 was characterized by frequent CDKN2A silencing, TP53 and TERT promoter mutations, CTNNB1 mutations and not frequent ethnicity; the cluster 3 was characterized by elevated hypermethylation, occurrence of IDH1/2 mutations and of a peculiar hypermethylation profile, rare CDKN2A silencing and TERT promoter and CTNNB1 mutations and frequent Asian ethnicity; cluster 4 was characterized by high hypermethylation, very frequent TERT promoter mutations and CDKN2A silencing, frequent CTNNB1 mutations, rare Asian ethnicity and frequent HCV [8].

Fujimoto and coworkers performed a comprehensive analysis of somatic genetic abnormalities in a large set of Japanese HCC patients and identified new driver genes for liver cancer, such as chromatin regulators (ASH1L and SETDB1), nuclear receptor signaling genes (TBL1XR1, NCOR1 and ESRRG), DNA-repair genes (NSMCE2 and MACROD2) and liver metabolism genes (HNF4A and G6PC) [9]. Interestingly, these authors observed also recurrently mutated non-coding elements, in addition to TERT promoter; particularly, six long intergenic noncoding RNA (lincRNA) genes, including NEAT1 and MALAT1, ten gene promoters including TFP12, MED16 and WDR74 and nine UTR, including BCL6 and AFF4, were identified as regions frequently mutated [9]. Finally, it was observed a relationship between gene expression and structural variations: thus, the expression level of oncogenes, such as CCND1, CBL and MET is increased, while the expression level of tumor-suppressor genes (CDKN2A, APC and AER1) is decreased, suggesting that mutational structural events contribute to hepatocarcinogenesis [9].

TP53-mutated HCCs have a poor prognosis compared with HCCs with WT TP53. A recent study showed that TP53-mutated HCCs become addicted to MYC stabilization via a mechanism involving Aurora Kinase A (AURKA): this MYC stabilization enables the tumor cells to overcome a latent G2/M cell cycle block, that is mediated by AURKA and p19^ARF^ [10]. This peculiar mechanism renders TP53-mutated HCCs sensitive to AURKA inhibitors, thus suggesting a therapeutic strategy for this subgroup of human HCCs [10].

Along with mutations, DNA copy alterations are also frequent genetic events in HCCs. Genomic deletions and gains at the level of various chromosome regions have been described: focal amplifications affect 6p21 (locus for VEGFA) and 11q13 (loci for FGF3/4/19/CCND1); recurrent homozygous deletions involved several genes relevant for cancer cell biology, such as AXIN1, CDKN2A/CDKN2B, IRF2, MAP2K3, PTEN, RB1. A copy number gain at 1q is one of the most frequently detected alterations in HCC and is an early genomic event in the development of HCC. Chromosome 8q is the second most frequently amplified region in HCC. The loss of heterozygosity at 4q is strongly correlated with increase in alpha-fetoprotein levels in HCC and is associated with poorly differentiated tumors. Finally, loss of heterozygosity on chromosome 8 is a frequent alteration in HCC, found in highly dysplastic nodules and, therefore, considered as an early alteration during hepatocarcinogenesis. The frequent copy number alterations observed in HCC suggests that genomic instability is an important driving component in the development of this tumor [11].

Some studies have explored the sequential evolution of the mutational landscape of liver cancer comparing these mutations in early tumor lesions and in advanced HCC. The large majority of HCCs develop from a pre-existing liver cirrhosis, which represents the “soil” for HCC growth. Thus, many liver cancers follow a pattern of pathologic evolution involving multistep processes, starting from cirrhosis to low-grade dysplastic nodules (LGDN), high-grade dysplastic nodules (HGDN), early HCC, progressed HCC and, finally, to advanced HCC. Early dysplastic nodules present a genome with a very limited genetic variation (a mean of five coding single-nucleotide mutations), while advanced HCC a heterogeneous genome with a range of 72–182 mutations [12]. As above mentioned, activating TERT mutations are very frequent in HCCs; these mutations are required to confer unlimited proliferative capacity. Several arguments support that TERT promoter mutations are an early transforming event. In fact, a progressive increase in the frequency of genetic alterations in the TERT locus from 6% in low-grade dysplastic nodules, to 19% in high-grade dysplastic nodules, to 61% in early HCCs [13]. In line with these observations, Nault and coworkers reported TERT promoter mutations in 54% of HCCs and 25% of cirrhotic pre-neoplastic nodules, thus suggesting that this genetic abnormality could represent one of the earliest recurrent genetic events occurring in cancer liver development [14].

In contrast, acquisition of genomic diversity through mutations of tumor suppressor genes and oncogenes, such as TP53, ARID1A, CTNNB1, occurred only at later stages of tumor progression. Recent studies provided evidence that, although HCC occurs following cirrhosis in most cases, a subset of HCCs may derive from the malignant transformation of hepatocellular adenomas (HCAs). Four major subgroups of HCA have been identified, defined by: (a) mutations inactivating HNF1A (H-HCA) occurring in 35% of the HCAs; (b) activation of β-catenin by mutations in exon 3 (bHCA), occurring in 15% of the HCAs; (c) inflammatory phenotype (i-HCA) with STAT3 activation, related in 60% of cases to IL6ST somatic mutations inactivating gp130 and more rarely to STAT3 or GNAS mutations, and occurring in 50% of the HCAs; (d) a remaining 10% of unclassified HCAa (U-HCA) [15]. Furthermore, about 10% of I-HCAs display mutation of the non-receptor tyrosine kinase Fyn-Related Kinase (FRK) [15]. In HCAs, CNNTB1 predisposes to transformation of HCAs in HCCs and TERT mutations are required as second molecular hit for a complete malignant transformation of HCCs [16]. Some patients develop HCC in a non-cirrhotic liver. Recent studies have shown in some of the patients the occurrence of etiological events related to HCC development. Thus, Nault and coworkers have reported in some of these patients the clonal integration of adeno-associated virus type 2 (AAV2) at the level of some cancer driver genes, including CCNA2 (cyclin 2), TERT, CCNE1 (cyclin E1), TNSF10 and KMT2B, inducing overexpression of the target genes [17]. According to these observations it was suggested a pathogenetic role of AAV2 in some HCC [17]. More recently, additional subgroups of HCAs have been identified and, particularly: a subgroup, characterized by weak β-catenin activation, associated with β-catenin exon 7 or 8 mutations, occurring in about 3% of cases; a subgroup, characterized by Sonic Hedgehog activation, associated with the INBHE/GLI1 fusion, occurring in about 4% of cases [18]. Molecular subtypes associated with β-catenin and Sonic-Hedgehog activation were associated with malignant transformation and bleeding, respectively [18].

Several studies have explored the occurrence of HCC intra-tumor heterogeneity. In this context, initial studies carried out in small HCCs have provided evidence about a consistent degree of intra-tumor heterogeneity with respect to histologic differentiation grade and proliferation activity: 64% of intra-tumor heterogeneity was observed for HCCs measuring 3 to 5 com in diameter and 25% to 47% of HCC smaller than 2 cm [19]. Recently, Friemel and coworkers reported the analysis of various tumor-related parameters (cell and tissue morphology, expression of various liver cell markers including CK7, CD44, alpha-fetoprotein, EpCAM and glutamine synthetase and mutations of TP53 and CTNNB1) in 120 different tumor areas derived from 23 HCCs. The results of this analysis showed 26% of heterogeneity at morphological level, 39% of heterogeneity at immunohistochemical level and 22% of heterogeneity at mutational level [20]. According to these findings it was concluded that HCC heterogeneity represents an important challenge for an optimal classification of this tumor and may considerably contribute to treatment failure and drug resistance [20]. Other recent studies have analyzed the intra-tumor heterogeneity of HCCs, providing evidence that this heterogeneity is highly variable from one patient to another: ubiquitous mutations varied from 8% to 97% among patients [21]. Branched tumor evolution was clearly detected in HCCs. According to these observations it was concluded that sequence analysis of a single lesion cannot completely characterize the genomic features in most of HCC patients [21]. It is important that multifocality in HCCs may originate from two different mechanisms: thus, multifocality may develop metachronously ad intrahepatic metastases (IMs) of primary cancer or synchronously as multifocal tumors (MTs) through a process of synchronous and independent tumor formation. The intra-tumor heterogeneity is higher in HCCs with MTs than in those with IMs [22].

Recently, Torrecilla and coworkers reported a fundamental analysis of tumor heterogeneity, coupled with the multi-step process of hepatocarcinogenesis [23]. Through the analysis of early lesions [dysplastic nodules (DNs) and small HCCs], trunk drivers were identified, corresponding in 51% of cases in TERT, TP53 and CTNNB1 mutations [23]. About 90% of the mutations in these genes were ubiquitous in different regions of large tumors and this finding strongly support that they are trunk events [23]. In multinodular HCCs, 35% patients displayed IMs; 85% of mutations in TERT, TP53 and CTNNB1 were retained in primary and metastatic tumors [23].

The observed intratumor heterogeneity in tumor livers has major implications for diagnosis and therapy of HCC. In fact, the observed intra-tumorigenic heterogeneity has wide implications in HCC therapy because the different clonal subpopulations within a single tumor may exhibit a differential sensitivity to drugs and may be responsible at large extent for drug resistance.

The development of molecular studies represented a fundamental tool to improve our understanding on the driver mechanisms involved in liver cancer development and provided also a sound and scientific basis to propose a classification of these tumors suitable for therapeutic approaches of targeted therapy. These studies have led to propose a classification of HCCs in three classes: a WNT subclass involving about 25% of HCC cases and characterized by recurrent CTNNB1 mutations and HCV etiology; a proliferation subclass involving about 50% of HCC cases and subdivided into two subgroups (S1-TGF-beta and S2-EpCAM-positive); an inflammation/interferon subclass involving about 25% of HCC cases [24]. The HCC subgroup with a progenitor cell EpCAM2 signature is associated with a poor prognosis. The HCC tumor subclasses identified by this molecular classification are not selectively responsive to specific targeted therapies [24].

Various studies have identified HCC molecular signatures based on the profile of mRNA expression. mRNA signatures indicating a pattern of gene expression typically associated with progenitor cells or cholangiocytes (i.e., EpCAM signature 3, CK19 signature) [25]. In line with these observations, other recent studies confirmed that Keratin19 (K19)-positive HCCs had a poor prognosis compared to K19-negative tumors, both in terms of relapse-free survival and overall survival, both for patients undergoing surgical resection and liver transplantation [26]. This study showed also that K19-positive HCCs possess stem cell properties [26]. The progenitor cell signature is also a useful prognostic marker to predict the outcome of HCC patients undergoing liver transplantation: in fact, the presence of progenitor cell markers was associated with a 5-year recurrence of 53% and a survival of 45%, while the absence of these markers was associated with 5-year recurrence of 19% and a survival of 67% [27].

A recent study showed that a 5-gene score signature based on the assessment of the expression of KRT19, HN1, RAMP3, RAN and TAF9, accurately predicted overall survival in four independent cohorts of Caucasian and Asian patients [28]. Other studies have shown that gene expression profiles of the surrounding non-tumoral liver tissue were highly correlated with HCC patient’s survival [29]. The combination of clinical, pathology and gene expression data derived from both tumor and peritumoral tissues allows a good prediction of recurrence of HCCs [28]. In addition to gene expression studies, recent evidences suggest that single genetic abnormalities, such as CDKN2A alterations and FGF-CCND1 amplification, have a negative prognostic impact [6].

Interestingly, a recent study provided evidence about a good correspondence between histological subtypes of HCCs and gene mutations: CTNNB1-mutated HCCs were large, well-differentiated, cholestatic, with microreticular and pseudoglandular histological structures; TP53-mutated HCCs were poorly differentiated with a compact pattern, multinucleated and pleomorphic cells and frequent vascular infiltration [30].On the other hand, the scirrhous subtype was associated with TCS1/TCS2 mutations, epithelial-to-mesenchymal transition and a progenitor expression profile; the steato-hepatitic subtype showed frequent IL-6/JAK/STAT activation, without CTNNB1, TP53 and TERT alterations; finally, the macrotrabecular-massive subtype was associated with high alpha-fetoprotein level, TP53 mutations, FGF19 amplifications, vascular invasion and poor survival [30].

**Figure 2:**
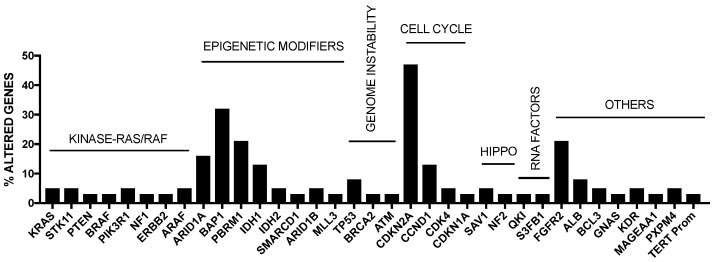
Frequent somatic alterations observed in cholangiocarcinoma (CCA). The data were reported by the TCGA in 38 CCA patients [29]. The altered genes were subdivided into different groups according to their function.

As mentioned above, intrahepatic cholangiocarcinoma (iCCA) is the second most common liver cancer after HCC and accounts for about 5–10% of all primary liver cancers. iCCA derives from the malignant transformation of cholangiocytes and must be distinguished from extrahepatic biliary cancers (eCCA) originating in large extrahepatic ducts. The highest incidence of iCCA was observed in Southeast Asia (particularly in Thailand). The most prevalent risk factor identified for HCC, such as cirrhosis and chronic hepatitis B and C infections, have been associated with iCCA, but not with eCCA. Studies carried out in the last years have defined the mutational spectrum of iCCA, showing a complex and heterogeneous pattern of genetic alterations. Some somatic mutations were recurrent in iCCAs, including KRAS (19%), TP53 (16%), IDH1/2 (15%), ARID1A (13%), BAP1 (11%), BRAF (5%) and EGFR (3%); furthermore, FGFR2 fusions are also frequent (23%) [31] (Figure 2).

**Figure 3:**
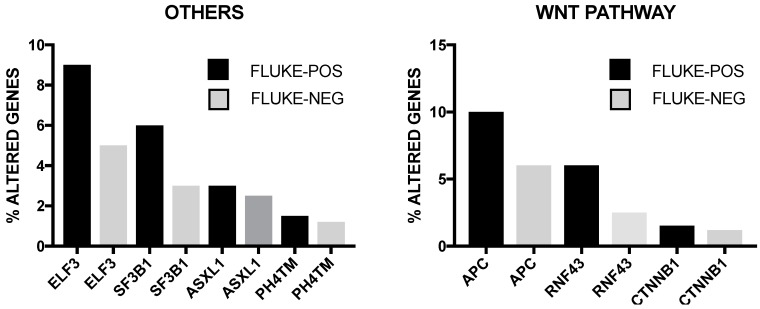

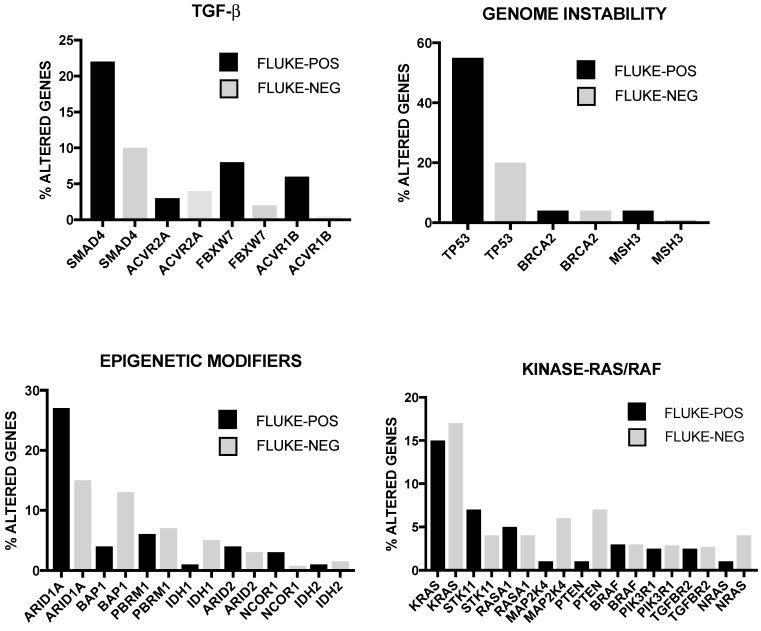
Frequently mutated genes in CCAs, subdivided into fluke-positive and fluke-negative patients. The data were based on the analysis of 489 CCAs and were reprinted from Jusakul et al. [34].

The incidence of some of these genetic alterations is greatly influenced by etiological factors related to this tumor. In endemic areas (i.e., Thainlandia) liver fluke infections by *Opisthorchis viverrini* (*O. viverrini*) and *Clonorchis sineusis*, represent the major risk for developing iCCA. Chan-on and coworkers have explored the mutational landscape of Asian CCAs, distinguished according to infections by *O. viverrini*: TP53 and SMAD4 mutations were more frequent among *O. viverrini* CCA (45% and 16%, respectively), compared to non-*O. viverrini* CCAs (7% and 0%, respectively); on the other hand, BAP1 and IDH2 mutations were less frequent among *O. viverrini* CCAs (3.2% and 3.2%, respectively), compared to non-*O. viverrini* CCAs (22.2% and 22.2%, respectively) [32] (Figure 3). These findings indicate that different causative etiologies induce distinct somatic alterations in CCAs [32]. Other studies have confirmed the frequent occurrence in iCCAs of inactivating mutations in various chromatin-remodeling genes (including BAP1, ARID1A and PBRM1): a mutation of one of these genes occurs almost in half of iCCA patients; in addition, mutations of the IDH1 and IDH2 genes were observed in about 20% of iCCA patients and their presence was associated with negative prognosis [33]. IDH mutant alleles observed in ICC (IDH1^R132K/S^) are different from those found in glioma and acute myeloid leukemia [34]. Integrative genomic analysis showed that IDH-mutant iCCAa display unique features, consisting of distinct mRNA, copy number and DNA methylation features; high mitochondrial and low chromatin modifier gene expression; methylation of the ARID1A promoter, with consequent ARID1A low expression [34].

Fujimoto and coworkers have performed whole-genome sequencing analysis on liver cancers displaying biliary phenotype (iCAA and combined hepatocellular cholangiocarcinomas) and have shown that the genetic alterations of cancers developing in chronic hepatitis liver overlapped with those of HCCs, while those of hepatitis-negative tumors diverged [35]. Importantly, the frequencies of KRAS and IDH mutations, associated with a negative disease-free survival, were clearly higher in hepatitis negative cholangiocarcinomas [31]. Recent studies have shown the occurrence of recurrent FGFR2 fusion events in iCCA patients (16% of patients); FGFR2 fusions are very rare in other primary liver tumors, being virtually absent in HCCs [36]. The most frequent FGFR2 fusion leads to the formation of the FGFR2-PPHLN1 fusion protein, possessing both transforming and oncogenic activities and inhibible by FGFR2 inhibitors [36]. Interestingly, in this study it was reported also frequent (11%) damaging mutations of the ARAF oncogene [36]. A significant correlation between FGFR2 fusions and KRAS mutations and signaling pathway activation was observed, thus suggesting a possible cooperative interaction in driving iCCA generation [36]. Studies carried out on large cohorts of Japanese patients suggest an association between FGFR2 fusions and viral hepatitis [37]. Since FGFR2 is targetable using specific FGFR2 inhibitors or multikinase inhibitors, clinical trials using these drugs are currently being investigated in iCCA patients harboring FGFR2 fusions.

Whole transcriptome analysis has shown the existence of two iCCA subclasses: one, characterized by a proliferation pattern, defining tumors with activation of oncogenic signaling pathways, including RAS/MAPK, MET and EGFR and poor prognosis; another characterized by an inflammation pattern, defining tumors with cytokine-related pathways, STAT3 activation and better prognosis [38]. A recent integrative genetic analysis of 489 CCAs proposed a classification for these tumors into four clusters [39]. Cluster 1 comprised mostly fluke-positive tumors, with enrichment of ARID1/A and BRCA1/2 mutations and high level of mutations in genes with histone lysine 3 trimethylation in their promoter. Cluster 2 was characterized by fluke-negative tumors, with upregulated CTNNB1, WNT5B and AKT1 expression and downregulation of genes involving EIF translation initiation factors [39]. Both clusters 1 and 2 were enriched in TP53 mutations and ERBB2 amplifications. Clusters 3 and 4 included the large majority of fluke-negative tumors. Cluster 3 was characterized by frequent copy number alterations, immune cell infiltration and upregulation of immune checkpoint genes [39]. Cluster 4 was characterized by BAP1, IDH 1 and IDH2 mutations and FGF alterations [39]. Interestingly, clusters 1 and 2 were enriched in extrahepatic tumors, while clusters 3 and 4 were composed most entirely by intrahepatic tumors [39]. BAP1 and KRAS were more frequently mutated in intrahepatic cases. At the clinical level, patients in clusters 3 and 4 had a better overall survival, compared to clusters 1 and 2. Another recent study based on genomic, transcriptomic and metabolomics analyses allowed to classify CCAs into four subgroups. The most common subtypes, C1 and C2, exhibited several distinct peculiarities. The ICC-C1 subtype was enriched for mitotic checkpoint signaling pathways (suggesting a high chromosome instability), frequent TP53, KRAS, MYC and GNAS mutations and display PLK1 and ECT2 as key drivers; the ICC-C2 subtype is linked to obesity, T cell proliferation, and bile acid metabolism [40].

iCCAs must be distinguished from eCCAs, not only for their different anatomical location, but also for some different etiological and molecular features. Thus, eCCAs are mainly related in their development to biliary lithiasis. Furthermore, at molecular level, TP53 mutations are frequent (26%), as well as KRAS (42–47%) and ARID1A (14%) mutations and 17q11 amplification involving ERBB2 (18%), while are rare IDH1/2 (2%), BAP1 (2%) and BRAF (0%) mutations. Furthermore, FGFR fusions are absent in eCCAs. Interestingly, Simbolo and coworkers have determined the mutational profiling of various types of biliary cancers, including iCCA, eCCA and gallbladder carcinomas (GBC) showing that IDH1/2 and BAP1 mutations were typically associated with iCCA, while KRAS and TP53 were most frequent in eCCA and GBC [41]. Some studies distinguish eCCAs into perihilar (pCCA) and distal (dCCA)/ampullary carcinoma: pCCAs are characterized by frequent fusion events of the Protein Kinase A components and mitochondrial synthase, α subunit (ATP1B) determining MAPK activation, ELF3 mutations and ARID1B mutations [42]; dCCAs and ampullary carcinomas display many molecular abnormalities similar to dCCAs, with frequent mutations at the level of TP53, KRAS, APC, CTNNB1, SMAD4, ARID2, ELF3, GNAS, ERBB2 and ERBB3 genes [43,44]. Two types of ampullary carcinomas have been identified: the intestinal-type is preferentially characterized by gene alterations at the level of the WNT signaling pathway, with the pancreatobiliary type predominantly harbor p53-Rb and RTK-RAS signaling alterations [44].

Fibrolamellar hepatocellular carcinoma (FLHCC) is a rare liver cancer usually occurring in adolescent and young adults, characterized at the histological level by the presence of lamellar fibrous bands composed of type I, III and V collagen, running through the tumoral tissue. At variance with HCC, FLHCC is usually not preceded by viral hepatitis and is associated with increased alpha fetoprotein levels or altered p53 or beta-catenin. The cellular origin of FLHCC is unclear in that a neuroendocrine origin was postulated based on increased expression of PCSK1, NTS, DNER and CALCA proteins [45,46], while a biliary tree origin was supported by increased levels of EpCAM, mCEA, CA 19-9, EMA and CK7 [47]. A recent study identified a peculiar genetic abnormality in FLHCC tumors consisting in a ≅ 400-kb deletion in chromosome 19, which determined the formation of a chimeric gene involving the first exon of the DNAJB1 gene and all but the first exon of the PRKACA gene: this gene encodes a chimeric transcript and a chimeric protein endowed with full catalytic activity of PKA [48]. The DNAJB1-PRKACA fusion transcript is strictly specific for FLHCC and was not observed in other hepatic tumors, including scirrhous hepatocellular carcinomas resembling FLHCCs [49]. The DNAJB1-PRKACA fusion is observed in the large majority of FLHCCs. Another gene fusion event, a translocation between the CLPTM1L and GLIS3 genes, generating a chimeric transcript which promotes cancer phenotypes in HCC cell lines, was observed in FLHCC [49]. Whole genome sequencing of 20 FLHCC tumors showed, aside from the deletion of chromosome 19 determining the DNAJB1-PRKACA fusion protein, there are no recurrent structural variations that contribute significantly to the tumor genotype [50]. FLHCCs possess a relatively stable genome, with a single recurrent deletion found in virtually all patients and few additional mutations (the most frequently mutated genes were MUC4 and GOLGA6L2) [50]. The apparent lack of a second-hit mutation in the genome of FLHCCs supports the role of DNAJB1-PRACA fusion protein as a major driver of this tumor and as a key diagnostic and therapeutic target [50]. Importantly, the DNAJB1-PRKACA transcript was expressed 10-fold higher than the WT PRKACA transcript, resulting in clear overexpression of the mutant protein in tumors [51]. Consequently, FL-HCCs exhibited elevated cAMP-stimulated PKA activity, compared to normal livers, thus suggesting that aberrant PKA signaling contributes to liver tumorigenesis [51]. Experiments of overexpression of the DNAJB1-PRKACA fusion protein in cell lines indicate that this protein retains its kinase activity, but does not possess constitutive PKA activity, although the fusion protein displayed a clearly increased PKA activity when stimulated by cAMP [52].

Recent studies have characterized at molecular level a variant of FL-HCC, known as mixed fibrolamellar hepatocellular carcinoma (Mix-FL-HCC) and characterized by the presence of both pure FL-HCC and conventional HCC component within the same tumors [53]. In these rare tumors, the DNAJB1-PRKACA fusion transcript is expressed at high levels [53]. Importantly, the analysis of RNA-sequencing data from The Cancer Genome Atlas (TCGA) for more than 9000 tumors across about 30 cancer types showed that the DNAJB1-PRKACA fusion is specific for FLHCCs [54].

Two recent studies have explored the gene expression profile of FLHCCs. Thus, Simon and coworkers have determined the transcriptomic profile of FLHCC, comparing the tumor tissue with the adjacent normal liver tissue, showing that FLHCC has a unique gene expression profile. Thus, although some gene expression changes were like those observed in HCCs, the majority were different. In FLHCC there was increased expression of various oncogenes, including pathways found to be activated in breast cancer, involving ERBB2, Aurora kinase A, E2F3 and CYP19A1 [55]. Importantly, the pattern of altered gene expression observed in FLHCC does not match with that of transcription factors known to be phosphorylated by PKA [55]. In a second study Cornella and coworkers have performed whole transcriptome analysis on 58 FLHCCs [56]. Gene expression clustering provided evidence about three different classes of tumors: most of tumors pertains to the proliferation class (51%) and displays altered expression of genes involved in the control of proliferation; about 26% of the tumors corresponds to the inflammation class; finally, the remaining 23% of tumors pertains to the unannotated class and displays a gene expression signature not previously associated with liver tumors [56]. Furthermore, expression of genes regulating neuroendocrine function, as well as histologic markers of hepatocytes and cholangiocytes are expressed in all three classes. A recent study by Sorenson et al. [57] based on the analysis of 36 FLHCC patients by comparative genomic hybridization, showing frequent copy number alterations, dependent upon a high level of chromosome instability, with genomic deletions or amplifications more frequent in metastatic than in primary tumors [57]. Gains at 1q21-23 were the most frequently seen chromosomal alteration [57]. Transcriptional profiling showed that genes frequently overexpressed are classified as genes pertaining to the neuroendocrine family, in line with a previous report [57]. Furthermore, gene expression studies show a role for p53 inactivation and IGF2BP1 upregulation in FLHCC [57].

Hepatoblastoma (HB) is the most common pediatric liver malignancy: it accounts for about 1% of all pediatric tumors and about 100 new cases are diagnosed each year in the United States. HBs form a heterogeneous group of liver tumors originated from hepatoblasts, rapidly proliferation hepatic progenitor cells that in the developing liver give rise to mature hepatocyte and biliary cell progenies. HBs derive from the uncontrolled expansion of hepatoblasts arrested at various stages of differentiation. At histological level, HBs are classified as either wholly epithelial (about 65%) or mixed epithelial/mesenchymal tumors (about 35%), with a predominant epithelial component represented by embryonal and fetal hepatoblasts.

Sequencing studies in HBs have consistently shown a low rate of mutations, compared to adult liver tumors. The most frequent genetic abnormality observed in HB is represented by exon-3 deletions or missense mutations in gene coding for β-catenin (CTNNB1), a downstream effector of the Wnt signaling pathway, in up to 90% of HB cases [58,59,60]. Most CTNNB1 mutations in HB are interstitial deletions or missense point mutations in exon 3 of CTNNB1: these mutations alter the serine/threonine phosphorylation sites of β-catenin or a residue near to these phosphorylation sites, rendering the β-catenin protein not recognizable for degradation. More rarely, mutations in other components of the β-catenin degradation complex, including those affecting APC and AXIN2 have been reported [60]. Whole-exome sequencing studies have confirmed the frequent occurrence of CTNNB1 mutations in HBs. Thus, Eichenmuller and coworkers reported mutations in exon 3 of CTNNB1 in 12 of 15 cases [61]. Jia et al. in another study, carried out in six HBs, identified 24 somatic nonsynonymous mutations occurring at the level of 21 genes: among these mutations, they reported three novel mutations affecting CTNNB1 (G512V) and CAPRIN2 (R968H/S969C) genes in the Wnt pathway and mutations of genes involved in the ubiquitin ligase complex (KLHL22, RNF169, SPOP and TRPC4AP) [62]. These observations strongly support a role for Wnt activation in HB development. However, despite the inferred obligatory role for Wnt in HB, enforced hepatic expression of mutant β-catenin was not sufficient to induce liver cancer development [63,64]. According to the sequencing data, only the anti-oxidant response regulator, nuclear factor (erythroid-derived 2)-like (NFE2L2, also known as NRF2), the second most frequently mutated gene in HBs [61,62]. In addition to NRF2, MYC could represent a potentially important contributor to HB development because it was identified as a prominent oncogenic signature in aggressive human HB [65]. In line with these observations, neonatal mice co-expressing mutant β-catenin and c-Myc preferentially develop HBs over other hepatocellular tumors [66]. Furthermore, the chronic β-catenin activation in liver cells determines a condition of hepatic NRF2-dependent anti-oxidant signaling [66]. In line with these findings, about 50% of HB tumors display aberrant activation of either Myc or NRF2 [66]. According to these findings, it was proposed that β-catenin acts creating a pro-tumorigenic hepatic environment, in part through activation of NRF2 and, therefore, oxidative stress could represent a driving force of a subset of HBs [66].

Sumazin and coworkers have recently reported the genomic characterization of 88 HB patients. About 90% of these patients displayed CTNNB1 mutations, while NRF2 and TERT promoter mutations were reported only in a minority of patients (5% and 2.5%, respectively) [67]. The joint analysis of WES, transcriptomic and miR expression data showed a stratification into three molecular subtypes. High-risk tumors were characterized by increased activity of NRF2; high expression of alpha-fetoprotein, lin-28 homolog B, high mobility group AT-hook 2, spalt-like transcription factor 4; high expression of oncofetal proteins and stem cell markers. In contrast, low-risk tumors were characterized by low lin 28 homolog N and lethal-7 expression and high HNF1A activity [67].

Mixed HCC-iCCA is a rare tumor (<1% of all primary liver cancers), whose diagnosis is based on the histological finding of both HCC and iCCA features. According to the 2010 WHO classification, two different types of this tumor are observed: a classic type, characterized at histological level by areas of typical HCC and iCCA, with a transition area; a stem cell-like type. The stem cell type can be in turn subdivided is into three subtypes (typical, intermediate and cholangiocellular carcinoma) [68]. The analysis of few mixed HCC-CCA samples suggested that these tumors may display similarities with hCCs and iCCAs with stem cell features [69]. A comprehensive molecular characterization of 18 samples of mixed HCC and iCCA defined three molecular types: CLC, stem-cell and classical. The CLC subtype, is a biliary-derived tumor, classified on the basis of histology as a stem-cell subtype, exhibits a peculiar molecular profile with biliary traits, low level of chromosome instability, activation of TGF-β and inflammation-related signaling pathways and frequent TP53 and IDH1 mutations; the stem-cell tumors were characterized by positivity in most of cases for the transcription factor SALL4 (75%), enrichment for progenitor-like signatures, activation of oncogenic pathways (MYC, IGF1R, NOTCH), signatures of poor clinical outcome and frequent FGFR2 fusion, TP53 and BRAF mutations; the stem-cell tumor SALL4^−^ display chromosome instability and frequent TERT promoter and TP53 mutations and have hepatocyte-like gene expression profile; the classical tumors display biphenotypic features, frequent TERT promoter and TP53 mutations and display poor prognosis signatures [70].

The studies on the molecular abnormalities of liver cancers have clearly evidenced the involvement of several oncogenic pathways acting at various stages during the tumorigenic process. Particularly, these studies have supported the role of immortalization pathway (TERT mutations), cell cycle deregulation (CDKN2A, RB1, MYC, CCND1 alterations), genomic instability pathway (TP53, ATM and MDM4 alterations), WNT signaling pathway (CTNNB1, AXIN1 and APC mutations), oxidative stress pathway (ARID1A and ARID2 mutations) and growth factor signaling pathway (FGF9, MET9, RPSKA3, JAK1 and PIK3CA alterations) [4,71]. Other play a role in initiation and upon HBV integration and HCV core protein [4,71]. Many of the recurrent mutations observed at the level of various genes seem to play a relevant role in liver tumorigenesis: thus, some mutated genes, such as TERT, JAK1 and RPSK3 play a role in hepatoacarcinogenesis; other ones, like AXIN1, CTNNB1, NFE2L2 and KEAP1 play a role both in hepatocarcinogenesis and progression; finally, ARID1A and ARID2 mutations play a role in initiation and progression of HCC [11,72]. It is important to underline that, although TP53 mutations have been identified as one of the most frequent alterations in HCC, however, their role in hepatocarcinogenesis remains unclear (reviewed in [11,72]). It is important to point out that most recurrently altered genes in HCC (TERT, TP53, CTNNB1, ARID1A) are unactionable targets, while actionable targets are observed only among genes more rarely altered [73].

## 3. Molecular Classification of HCCs

The analysis of the main molecular events characterizing the development of HCCs has allowed a molecular classification of these tumors to be proposed, representing a good compromise between the analysis of gene expression profiling, the main biological properties of various HCC subtypes and their prognostic/clinical implications. Basically, two main molecular subtypes of HCCs have been described: one is defined as proliferation class and is characterized by an enrichment of molecular signals related to proliferation and progression in the cell cycle and is usually associated with a more aggressive phenotype; the other one is defined as nonproliferation class and is associated with the maintenance of molecular features resembling normal differentiated hepatocytes [74]. Particularly, the proliferation class is characterized by more aggressive clinical and biological features, higher incidence of tumor relapse and shorter survival, increased cellular proliferation, activation of pro-survival, anti-apoptotic pathways mediated by MET and E2F1, larger and less differentiated tumors, various types of genetic abnormalities associated with loss of TP53 function [75]. The proliferation class can be subdivided into two subtypes distinguished according to some molecular features: a S1 subclass showing higher TGF-β and cholangioma-like signature, while the S2 subclass displayed positivity for the stemness marker EPCAM, high alpha-fetoprotein, activation of IGF2 pathway and suppression of interferon target genes [75]. A part on non-proliferative HCCs are characterized by somatic mutations occurring at the level of exon 3 of CTNNB1 accompanied with induction of specific target genes, such as LGR5 and GLUL [75], but not with canonical WNT genes. It is of interest to note that canonical WNT target genes are more frequently activated in the HCC proliferative subtypes [75]. Gains in genes located at Chr. 11q13 (CCND1 and FGF19) are frequent in tumors from the proliferation subclass [3,76]. This molecular classification, if implemented with additional molecular markers, could be useful for the identification of HCC patients amenable to current clinical experimental trials of targeting therapy.

A recent study analyzed the prognostic impact of the expression level of DNA repair genes in HCCs (carrying the analysis in TCGA dataset and in 120 independent cases) showing the level of DNA repair genes inversely correlated with prognosis: thus, according to the level of DNA repair gene expression the HCC patients were divided into four groups [77]. Group 1 and 2, displaying the highest levels of expression of DNA reparation genes, exhibited the lowest overall survival and tumor-free survival; these two groups were characterized at molecular level by frequent mutations of TP53 and AXIN1 cluster genes [77]. In contrast, cluster 3 and 4 of patients displayed lower levels of expression of DNA reparation genes and exhibited longer overall survival and tumor-free progression; these tumors at molecular level were characterized by low frequency of TP53 cluster gene mutations and frequent mutations of the CTNNB1 cluster genes (particularly, group 3 of HCC tumors) [77].

**Figure 4:**
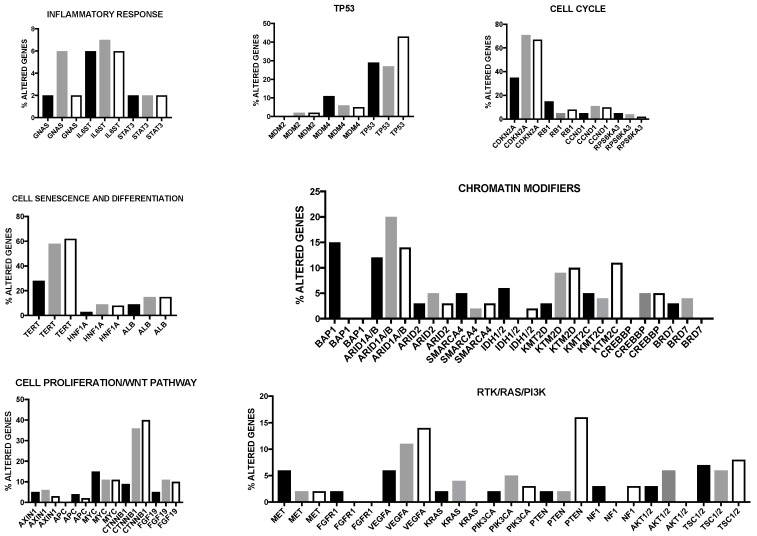
Integrated molecular analysis of somatic alterations in signaling pathways in HCC patients subdivided into three different clusters (cluster 1, black bars; cluster 2, gray bars; cluster 3, white bars). The data are reprinted from a recent study of the Cancer Genome Atlas Network [5]. Somatic alterations include mutations, copy number alterations and epigenetic silencing of CDKN2A.

An integrative extensive genetic analysis recently allowed the classification of HCCs into three different clusters (Figure 4). The cluster 1 was associated with a prevalent Asian ethnicity, high tumor grade, low differentiation score, low frequency of CDKN2A silencing, TERT promoter mutation and CTNNB1 mutation, high miR-181a expression and overexpression of proliferation marker genes. The cluster 2 and cluster 3 displayed a high frequency of CDKN2A inactivation by DNA hypermethylation, of TERT promoter and CTNNB1 mutations and enrichment of HNF1A mutation [8]. At the clinical level, cluster 2 was associated with low-grade tumors, while cluster 3 was characterized by a high grade of chromosome instability, high frequency of TP53 mutations and a distinct chromosome abnormality (17p loss) [8]. Among these three clusters, cluster 1 had a worse prognosis [8]. In addition to this stratification for multiparameter clustering, this integrate genetic analysis allowed to identify two HCC subgroups characterized by IDH mutations and by low p53 expression. IDH1 mutations (R132C and R132G) and IDH2 mutations (R172K and R172S) in HCCs were associated with tumors with a typical HCC histology. Interestingly, IDH-mutant HCCs display a typical gene expression pattern, observed also in some HCC IDH-wild type and resembling a hepatic stem-cell [78]. IDH-mutant and IDH-like HCCs were associated with a negative prognosis [8]. Interestingly, miR-122, the micro RNA most abundantly expressed in liver is downmodulated in IDH-mutant HCCs [8]. Mutations involving P53 are frequent and are associated with low P53 function, as evaluated by assessment of P53 transcriptional target expression; however, the low P53 target expression HCC group included also some P53-WT tumors: in these cases, the low P53 expression was ascribed to overexpression by gene amplification of MDM4, a P53 inhibitory protein. HCCs with low P53 target expression exhibited significant tendency to increased copy number alterations, higher pathologic grade, reduced expression of hepatocyte differentiation marker genes and increased risk of tumor recurrence [8].

A recent study showed that about 25% of HCCs display markers of an inflammatory response, with high expression of programmed death-ligand 1 (PDL1) and programmed cell death 1 (PD1); this group of HCCs was called an immune-specific class and was subdivided into two different subtypes characterized either by markers of an adaptive T-cell response or exhausted immune response [79]. According to these findings, it was suggested that HCCs might be susceptible to treatment with immune check inhibitors. In line with this hypothesis, a phase 1–2 study (CheckMate 040) of nivolumab (Opdivo^®^, a PD1 inhibitor) in patients with advanced HCC, with or without chronic viral hepatitis, previously treated or not with sorafenib, showed a 20% of objective response rate [80].

## 4. Cell Origin of Liver Cancer

Many studies carried out in these last years have attempted to define the cellular elements whose malignant transformation generates liver cancers. Three distinct cellular elements are present in adult liver: hepatocytes and adult stem and progenitor cells, known as oval cells.

It is unclear whether liver progenitors are derived from biliary-like stem cells that acquire hepatocyte phenotype and functions or from hepatocytes losing hepatocytes functions. Tarlow and coworkers showed that clonally-traced Sox9^+^ progenitors give only a marginal contribution to mouse liver regeneration under normal conditions and following oval cell injuries [81]. Lineage-tracing experiments have confirmed these conclusions providing evidences against a stem cell origin of new hepatocytes in common mouse models of chronic liver injury, thus indicating a very limited capacity of non-hepatocyte progenitors to contribute to liver regeneration [82,83].

In contrast, numerous studies indicate that hepatocytes undergo a reprogrammation involving their “transdifferentiation” into ductal biliary cells in various experimental models involving either cell injury [84,85] or the enforced expression of either NOTCH [86] or HIPPO pathways [87].

Other recent studies based on hepatocyte transplantation models following acute hepato-biliary injury provided evidence about a major contribution of mature hepatocytes to biliary regeneration [88].

Importantly, a recent study provided evidence that a population of periportal hepatocytes, located in the portal triads of normal livers and expressing low Sox9 levels and other bile-enriched genes (for these reasons called hybrid hepatocytes), are capable of extensive proliferation and of replenishment of the hepatic mass following chronic hepatic injury and this represents a unique cellular reserve to regenerate damaged liver [89]. According to these findings it was concluded that regeneration occurring after most types of liver injury or after partial hepatectomy is achieved through the hepatocyte pool, without a major contribution of progenitor cells [90]. Wang et al. used a genetic lineage tracing of Axin2^+^ cells (Axin2 is a universal transcriptional target of β-catenin) and using this approach have identified a unique population of Wnt-responsive cells that surround the central vein: these cells are diploid and express early progenitor cell markers, self-renew and give rise to polyploid mature hepatocytes [91].

A recent study provided evidence that hepatocyte-to-duct conversion is reversible. Thus, Tarlow and coworkers have used a chimeric lineage tracing system to show that hepatocytes contribute to the liver progenitor pool: in chronically injured livers, some hepatocytes are able to generate bipotential liver progenitors able to undergo ductal differentiation; hepatocyte-derived proliferative ducts are able to differentiate back into hepatocytes upon cessation of injury [92].

Other studies suggest a possible involvement of biliary-derived stem/progenitor cells in liver regeneration. These studies were originated from the observation that ductular reactions (DRs), corresponding to cellular reactions of ductular phenotype at the portal-parenchymal interface, are frequently observed in response to acute or chronic injury in the large majority of human liver diseases. It is commonly accepted that DRs derive from the biliary compartment or their outermost terminals, the canals of Hering and contain progenitors that normally reside in a quiescent state in the canals of Hering (commonly called as liver progenitor cells or adult hepatic stem cells). These putative progenitors may act as an adult emergency reservoir able to generate biliary ductal cells and hepatocytes in conditions of acute or chronic liver injury compromising the duplication capacity of hepatocytes. The biliary ductal differentiation of DR cells represents their canonical differentiation capacity, promoted juxtacrine NOTCH activation, while the paracrine activation of beta-catenin by WNT proteins may modify the differentiation capacity of DR cells, inhibiting biliary differentiation and promoting hepatocytic differentiation [93].

However, other studies have shown that in some models of chronic liver injury or when the proliferative capacity of hepatocytes is inhibited, a reserve cell compartment of liver progenitor cells (oval cells) undergo proliferation and actively generate hepatocytes [94]. In line with these findings other studies suggest that LGR5+ hepatic progenitors could generate hepatocytes. These studies have initially shown that single mouse LGR5+ stem cells can be expanded in vitro as epithelial organoids and are able to differentiate into hepatocytes both in vitro and in vivo [95]. In a subsequent study the same authors showed that bile duct-derived bipotent progenitor cells expanded in vitro differentiate into mature functional hepatocytes: these progenitors can be isolated from ductal EpCAM^+^ ductal cells, but not from hepatocytes (EpCAM^−^) [96]. Biliary duct cells derived from murine or human liver, when cultured in medium containing FGF, HGF and Wnt Ligands, undergo the generation of long-term expanding, 3D organoid cultures able to both self-renew and generate hepatocytic and ductal cells in vitro [96]. Raven and coworkers have confirmed that the inhibition of hepatocyte proliferation during liver injury triggers a cholangiocyte reaction, with 25% of hepatocytes being derived from a non-hepatocyte, cholangiocyte origin [97]. According to these findings it was concluded that cholangiocytes can act as facultative liver stem cells during impaired hepatocyte regeneration [97].

In addition to these cell populations, recent studies indicate that a liver-resident population of mesenchymal stem cells, hepatic stellate cells, may function as multipotent progenitor cells capable of generating both functional hepatocytes and cholangiocytes [98]. Hepatic stellate cells reside between the hepatocytes and small blood vessels in the liver. These cells are characterized by the presence of lipid droplets and thin protrusions extending around the blood vessels [99]. Their activation in damaged liver leads to secretion of collagen and formation of scar tissue, leading to chronic fibrosis or cirrhosis [99]. Hepatic stellate cells are retinoid-storing cells with a non-proliferative, quiescent phenotype: these cells store particularly high levels of retinoids, mainly as retinyl palmitate [99]. Following liver injury, hepatic stellate cells become activated, transdifferentiating from vitamin-A-storing cells to myofibroblasts and, through this mechanism, can contribute to fibrogenesis in chronic liver disease [99]. Hepatic stellate cells differentiate into hepatocytes following adequate growth factor stimulation. A recent study showed that bile acids induce hepatic differentiation of mesenchymal hepatic stellate cells through a molecular mechanism involving the forsenoid X receptor and transmembrane G-protein-coupled bile acid receptor 5 [100].

In conclusion, all these studies have underlined the high plasticity of resident adult hepatocyte and ductal liver populations. Thus, diploid hepatocytes, differentiated parenchymal cells and various populations of stem/progenitor cells have all been contributed to time homeostasis under steady-state and regeneration conditions.

Studies of hepatocarcinogenesis are of fundamental importance to understand the molecular mechanisms of liver cancer development and to determine the cellular origin of liver cancers. These models were based on the introduction into liver cells of some mutant genes or of some genes abnormally expressed. Usually transgenic or knockout are the most used methods to develop cancer mouse models. Particularly, genetically engineered mice, including transgenic or knockout mice, are required to demonstrate the oncogenic or tumor-suppressor potential of target genes and to show how these genes contribute to tumor initiation and development. The development of a new method of gene delivery into hepatocytes, hydrodynamic gene delivery, was of fundamental importance in the development of hepatocarcinogenesis models. These methods combine hydrodynamic gene delivery and Sleeping Beauty-mediated somatic integration for long-term gene expression in mouse hepatocytes [101]. Using this methodology, it was provided evidence that various pathways are tumorigenic for hepatocytes, including p53, WNT-β-catenin, TGF-β, hepatocyte growth factor (HGF)-MET, IGF and EGFR [101]. In this context, particularly interesting are the studies carried out TP53. Initial studies in mouse models have shown that p53 inactivation is required for the maintenance of murine liver cancers in vivo. A recent study showed that loss of p53 favored the de-differentiation of hepatocytes into nestin-positive progenitors, which are poised to generate either HCCs or iCCs in response to lineage-specific mutations targeting WNT and NOTCH signaling, respectively [102]. Therefore, under normal conditions transcriptional repression of Nestin exerted by p53 restricts cellular plasticity and tumorigenesis in hepatocytes [102].

Other recent studies have shown that disruption of signaling pathways playing a key role in liver homeostasis (control of liver size) and differentiation status and plasticity of hepatocytes, such as Hippo-YAP and Neurofibromin 2, represents an important step in hepatocarcinogenesis and is able to induce HCC formation. In this context, particularly interesting are the studies related to the Hippo-YAP pathway. Hippo signaling is an important tumor suppressor mechanism in normal liver. Through a complex signaling pathway involving the STK3 and STK4 kinases, the Hippo pathway targets the transcriptional co-activators YAP and TAK, inducing their phosphorylation, leading to cytoplasmic retention and degradation. In adult mouse liver YAP overexpression determines the expansion of atypical ductal cells (ADC, resembling oval cells), hepatocyte proliferation, liver expansion and progression to HCC [103]; lineage tracing in transgenic YAP livers showed that ADCs are derived from the de-differentiation of hepatocytes [103].

These observations have indicated that defective Hippo signaling in hepatocytes determines HCC development. More recent studies have explored in more detail how YAP may act as a controller of hepatocyte cell fate acting as a rheostat, maintaining metabolic, differentiation and quiescence control in the hepatocytes [88]. YAP levels decrease or increase reprogram hepatocytes to different cell fates, through deregulation of HNF4A, CTNNB1 and E2F transcriptional programs, thus affecting hepatocyte proliferation and differentiation [103]. In this context, particularly interesting was the observation that targeting YAP expression with a specific small interfering RNA in HCC restores hepatocyte differentiation and induces tumor regression in a suitable mouse model [103]. In primary HCCs, YAP targets are enriched in an aggressive HCC subtype [103].

A recent study used a complementary fate-tracing approach to label three different cellular compartments, progenitor, biliary and hepatocytes, and explored their contribution to experimental hepatocarcinogenesis: both in genotoxic and genetic cancer models, HCCs arose exclusively from hepatocytes and not from progenitor/biliary compartment [104]. Within the tumors, cytokeratin-19 and alpha-fetoprotein-positive cells derive from hepatocytes [104]. These observations strongly support the idea that HCCs derive from malignant transformation of hepatocytes and a progenitor signature in liver cancers does not reflect progenitor origin, but de-differentiation of hepatocyte-derived tumor cells.

The studies on mouse models underline the plasticity of hepatocytes. Numerous experimental evidences indicate that hepatocytes, in response to acute or chronic biliary undergo a reprogramming of cell fate involving a shift from hepatocytes to cholangiocytes, through a NOTCH-mediated cell conversion process [105]. The direct role of NOTCH activation in this cell reprogramming event is directly supported by the observation that constitutive NOTCH signaling in hepatocytes or enforced co-activation of both NOTCH and AKT signaling by hydrodynamic gene delivery induced the generation of biliary lineage cells and elicited also the development of biliary tract cancers [106].

As above mentioned, a recent study provided evidence that in the presence of a chronic liver damage, hepatocyte-derived newly formed biliary duct cells retained a biologic memory of their initial hepatocytic differentiation status and return to the hepatocytic lineage after cessation of the liver injury [102]. These observations have stimulated the hypothesis that cholangiocarcinomas could derive from the malignant transformation of chronically injured hepatocytes when these cells have reached a trans-differentiation condition.

Although there is strong evidence that iCCA could derive from hepatocyte transformation via reprogrammation of these cells to biliary ductal cells, some studies have suggested a biliary duct origin for these tumors. Thus, Guest and coworkers have used a cholangiocyte-lineage system to target p53 loss to biliary epithelia and have observed the generation of labeled biliary lineage tumors in response to chronic liver injury [107]. As expected, activation of NOTCH signaling was observed in these tumors [107]. According to these observations, it was concluded that in the context of p53 loss and chronic inflammation, biliary epithelia are a target of malignant transformation and represent the origin of iCCAs [106]. This conclusion is also supported by a recent study showing in a mouse model of liver regeneration, where the E3 ubiquitin ligase MDM2 is inducibly deleted in hepatocytes with consequent and necrosis of hepatocytes, the massive activation of biliary-derived liver progenitors [108]. In transplantation assays, these progenitors regenerate both hepatocytes and biliary epithelia, thus indicating their multipotency [108].

The exploration of various mouse models of iCCA highlighted the importance of activation of WNT signaling and chronic inflammation in the development of iCCAs [109].

Other animal models of hepatocarcinogenesis suggest a progenitor cell origin of liver tumors. Thus, Chiba and coworkers have shown transduction of C cell-specific Moloney murine leukemia virus integration site 1 (Bmi 1) or mutant β-catenin into murine fetal hepatic stem/progenitor cells induced the stimulation of self-renewal and tumorigenicity, with formation of hepatic tumors of combined HCC-CCAs histology [110]. Moreover, another study showed the initiation of HCCs and hepatoblastomas in transgenic mice with hepatic stem/progenitor cells that overexpress β-catenin [111]. In other models, the development of HCCs or CCAs is observed in tumors originated from TP53-deficient or liver-specific p53-mutant murine hepatic progenitors transduced with c-Myc [112] or K-Ras [113].

Using human data from various hepatocarcinogenesis models, Tummala and coworkers reached the important conclusion that HCCs originate predominantly from hepatocytes and benign adenomas from hepatic progenitor cells [114]. Genetic lineage tracing experiments of HPCs and hepatocytes showed that in all animal models HCCs originate from hepatocytes. Mechanistically, signals originate from transformed hepatocytes, based on paracrine effects induced by α-ketoglutarate (maintaining HPCs in an undifferentiated state) and by galectin-3 (maintaining HPC stemness) induce the malignant transformation of HPCs into HCCs [114]. Genetic blockage of galectin-3 reduces HCC and its expression in HCC correlates with poor survival [114].

Other studies have defined the role of some genes, such as IDH1 and IDH2, frequently mutated in iCCAs in tumor development in mouse models. Particularly, these studies have shown that mutant IDH blocks liver progenitor cells from undergoing hepatocytic differentiation by a mechanism involving the production of 2-hydroxyglutarate and suppression of the transcription factor HNF-4α, a master regulator of hepatocyte lineage; in parallel, IDH causes an expansion of liver progenitor cells [115]. In these experimental systems IDH mutations cooperate with Ras mutations to drive both the initial and late events leading to iCCA development [115]. Additional studies support the hypothesis that the deregulation of other genes involved in cell metabolism could play a relevant role in hepatocarcinogenesis. Thus, it was recently shown that PKM2 loss leads to spontaneous development of HCC with high penetrance, accompanied by changes in systemic metabolism, characterized by altered systemic glucose homeostasis [116].

The above discussed results indicate that different cell types may be the initial targets of transforming events and contribute to liver cancer development. Most of these studies indicate the importance of cellular reprogramming of differentiated cells into a less differentiated, multipotent state, during the generation of liver cancers. In this context, it is particularly pertinent a recent study assessing the relative capacity of different hepatic cell lineages, including mouse primary hepatic progenitor cells, lineage-committed hepatoblasts and differentiated adult hepatocytes, to induce the formation of liver cancer by transgenic expression of HRAS and SV40 Large T antigen [117]. The results of this study provided evidence that any cell type at the level of the mouse hepatic cell lineages can undergo oncogenic transformation into a tumor-initiating cells able to generate a primary liver cancer, through activation of different cell pathways.

FGF19 is one of the most frequently amplified genes in HCC patients. Mice expressing a FGF19 transgene develop HCC [118]. FGF19 induces a non-cell autonomous program of activation of STAT3 signaling through induced IL-6 production in the hepatic microenvironment [118]. This conclusion is supported by the observation that IL-6 or STAT3 ablation inhibited FGF19-driven tumorigenesis [118].

## 5. Liver Cancer Stem Cells

Initial studies of characterization of liver cancer stem cells were based on the identification of the side population after staining with the DNA-binding dye Hoechst 33342: the side population purified from these tumors was enriched in cells displaying cancer stem cell properties [119,120]. Subsequent studies were focused on the attempt to identify a reliable membrane marker for liver cancer stem cells (CSCs); in this context, attention was focused on CD133. Initial studies were based on the identification and characterization of CD133^+^ cells in hepatocarcinoma cell lines. Thus, Suetsugu and coworkers reported in 2006 that CD133^+^ cells, isolated from the Huh7 cell line, display, when compared to their CD133^−^ counterpart a higher proliferative and tumorigenic capacity [121]. Furthermore, it was shown that the CD133^+^ fraction exhibits a more immature differentiation phenotype, compared to the CD133^−^ fraction. These initial observations suggested that in hepatocarcinoma CD133^+^ cells may represent a more immature tumor population, displaying a high tumorigenic capacity. These findings were confirmed in another hepatocarcinoma cell line, SMMC-7721, showing also the CD133^+^ cells isolated from this cell line display enhanced tumorigenicity in vivo when assayed by xenotransplantation into immunodeficient mice [122]. CD133^+^ and CD133^−^ cells were isolated from a panel of hepatocarcinoma cell lines, including HepG2, PLC 8024 and Huh78 and analyzed for their stem cell properties: CD133^+^ cells were shown to possess a greater colony-forming capacity in vitro, higher proliferative activity, greater ability to form tumors in vivo both using a subcutaneous and orthoptic tumor model and displayed also the capacity to differentiate into non-hepatocyte-like, angio-myogenic-like lineages [123]. The CD133^+^ cell population was found also to have enhanced expression of stem cell associated genes, such as BMi1, Sox2, Oct4, NOTCH, Nanog, Nestin, membrane transporters ABCG2 and ABCB1). Subsequent studies were focused to characterize CD133^+^ cells from primary human hepatocarcinomas, providing evidence that in primary tumor samples these cells account for 1.3 to 13.6% of the total tumor cell population, possess the capacity to form tumor spheroids composed by undifferentiated tumor cells and have a greater ability to form tumors of identical morphology compared to the parental tumors, when inoculated into immunodeficient mice [109]. Furthermore, the frequency of CD133^+^ cells into the primary tumor samples seems to have a negative impact on clinical outcome [124]. Interestingly, CD133^+^ cells were shown to overexpress miR-130b, a miR able to target and to lower the levels of p53-induced nuclear protein 1 (TP53/NP1): reducing TP53/NP1 levels into CD133^+^ cells, increased their tumorigenicity [124].

The identification of CD133^+^ cells as putative cancer stem cells of liver cancers prompted many studies attempting to characterize the biological properties of these cells and the mechanism through which these cells maintain and promote tumor growth. In this context, a useful approach consisted in analyzing the genome-wide microarray analysis the gene expression profiles of CD133^+^ cells compared to that of CD133^−^ cells isolated from both tumor cell lines and primary tumor samples: IL-8 and the IL-8 signaling network was found to be upregulated at the level of CD133^+^ cells [125]. IL-8 secretion by CD133_+_ cells is required for their self-renewal and for tumor initiation: inhibition of IL-8 secretion or blockade of its biological activity inhibited these activities of CD133^+^ cells [125]. IL-8, as well as SDF-1α production by CD133^+^ cells is stimulated by neurotensin; IL-8 released by CD133^+^ cells acts on these cells though an autocrine mechanism and induces the activation of the MAPK signaling pathway [125].

Other studies have explored the sensitivity of CD133 liver cancer cells to anticancer chemotherapy and to radiation therapy. Purified CD133^+^ cells, purified from hepatocarcinoma cell lines and from tumor xenografts, survive better than CD133^−^ counterpart to treatment to chemotherapeutic agents (doxorubicin and fluorouracil) [126]. The biochemical mechanisms mediating chemoresistance of these cells seem to involve AKT/PKB and Bcl-2 pathways, both hyperexpressed and activated in these cells [126]. Another study provided evidence that hypoxia activates both HIF-1α and AKT, contributing to maintain the chemoresistance of hepatic cancer progenitor cells; the hypoxia-mediated pathway involves also the production of PDGF-BB that, through an autocrine mechanism, contributes to AKT activation [127].

In parallel, other studies have addressed the problem of the radiosensitivity of liver cancer CD133^+^ cells, providing evidence that these cells contribute to the radioresistance of hepatocarcinomas. This conclusion was supported by the observation that liver CD133^+^ CSCs are more resistant to radiation-induced apoptosis than CD133^−^ cells; furthermore, these cells exhibited greater proliferation and tumor-initiating capacity in vivo, post-irradiation [128]. At least at some extent, the radioresistance and chemoresistance of CD133^+^ liver CSCs is related to their CD133 expression in that the downmodulation of this membrane antigen with a siRNA elicited both a decrease of their stemness properties and enhanced their chemosensitivity and radiosensitivity [129].

The expression of other membrane markers, in association with CD133, was explored, to try to better define the cancer stem cell population and, eventually, to identify subpopulations of these cells. Among these various membrane antigens, attention was given to the study of CD44. CD44 is a major adhesion molecule of the extracellular matrix, implicated in a wide range of biological processes; the CD44 antigen is produced by the cells under various isoforms, with the CD44s (CD44 standard) and the CD44v (CD44 variant) isoforms being the main isoforms. The CD44 was described as a stem cell marker of various cancer stem cell populations and was involved in tumor progression and metastasis. In hepatocellular carcinoma CD44s expression was related to regulation of the mesenchymal phenotype mediated by TGF-beta and its level of expression was associated with negative prognosis [130]. Using in combination the labeling of CD133 and CD44 antigens, CD133^+^CD44^+^ and CD133^+^CD44^−^ subpopulations have been identified. First, isolating these two subpopulations from various hepatocarcinoma cell lines it was possible to demonstrate that the CD133^+^CD44^+^ subpopulation was more tumorigenic than the CD133^+^CD44^−^ subpopulation; furthermore, the former one expressed more stem cell-associated markers, such as Bmi-1, and was more chemoresistant than the latter one [131]. In a second study, it was provided evidence that CD133^+^CD44^+^ subpopulation was associated with the capacity to form metastasis in the xenotransplantation assay in nude mice [128]. Finally, a link was observed in primary tumors between the number of CD133^+^CD44^+^ cells and portal vein metastases [132].

Using a rat model of hepatic carcinogenesis, Zheng and coworkers have shown that precancerous CD133^+^CD44^+^CD45^−^ cells, that formed a part of the hepatic oval cells, have stem cell properties (expand clonally, differentiate into two different hepatic lineages) [133]. Agents clinically used for the treatments of HCC, such as acyclic retinoids, suppress the expansion of these tumor progenitors [133].

A recent study explored the microenvironment factors that could promote the growth of HCC CD44^+^ cells. First, it was shown that tumor-associated macrophages (TAMs) promoted the expansion in vitro of CD44^+^ cells isolated from HCC primary tumors and from HCC cell lines; second, it was shown that TAMs produce IL6, which promotes the expansion of CD44^+^ cells and stimulates tumorigenesis; third, through blockade of IL6 signaling, it was provided evidence that IL6 was at large extent responsible for the tumor-promoting activity of TAMs [134].

The possible clinical impact of the level of CD133 expression in hepatocellular carcinoma was explored in the context of several clinical studies. Thus, Song and coworkers have explored the correlation between CD133 expression and clinico-pathological data and observed that: (a) increased CD133 expression levels correlate with tumor grade, stage disease and alpha-fetoprotein levels; (b) patients with increased CD133 levels had shorter overall survival and higher recurrence rates [128]. Recently, Ma and coworkers have performed a meta-analysis of all the data available in literature about the correlation between CD133 expression and various clinic-pathological parameters [135]. This analysis involved the evaluation of data obtained on 1344 hepatocellular carcinoma patients. These data showed that CD133 level of expression correlated with a poor histological grade, elevated alpha-fetoprotein levels, poor survival, but did not show significant relation with tumor stage, hepatitis and cirrhosis [136].

Other studies have led to the identification of various markers that could help to identify liver cancer stem cells. Lee and coworkers identified CD24, a mucin-like cell surface glycoprotein as marker of liver cancer stem cells. To do this study, these authors have used a peculiar strategy consisting in the isolation in xenotransplants of HCC of chemoresistant tumor cells [137]. The chemoresitant tumor cells displayed an enhanced CD24 expression. CD24 expression was observed in a minority of hepatocarcinoma cells in primary tumor specimens (ranging from 0% to 16%), while its expression was absent in nontumoral hepatic tissue [124]. CD24^+^ cells isolated from primary tumors displayed properties of cancer stem cells (as few as 500 of CD24^+^ cells are sufficient to induce tumor formation in immunodeficient mice) [137]. Importantly, CD24 expression at a large extent overlaps with CD133 and EpCAM expression on HCC. CD24 was not only a marker of HCC cancer stem cells, but exerted also a functional role in maintaining the stemness of these cells as suggested by CD24 knockdown experiments showing a reduced tumorigenic activity of these cells and a reduced expression of stemness genes involved in self-renewal, such as NANOG [137]. A recent study showed that CD24 expression on hepatocarcinoma cells is stimulated by Twist2, a basic helix-loop-helix transcription factor playing a key role in embryogenesis [138]. The stimulatory effect of Twist2 on CD24 expression is related to a transcriptional effect by direct binding to an E-box present in the CD24 gene promoter [138]. The stimulatory effect on CD24 expression is required for the stimulation of HCC stem cell self-renewal [138].

Other studies aimed to define EpCAM as a possible marker of liver CSCs. EpCAM is expressed on a subset of normal epithelia and overexpressed on malignant cells derived from a variety of malignant tumors; furthermore, EpCAM was particularly over-expressed on some populations of CSCs [139]. In this context, initial studies have shown that hepatocellular carcinomas may be subdivided into two different subgroups according to their positivity for EpCAM: EpCAM^+^ hepatocarcinomas displayed features typically observed at the level of hepatic progenitor cells, including the expression of a series of stem/progenitor cell markers, such as cytokeratin-19, c-kit and activated Wnt-beta catenin signaling; EpCAM^−^ hepatocarcinomas exhibited features more typical of mature hepatocytes [140]. EpCAM expression in hepatocarcinoma cells is induced by Wnt-beta catenin: this finding explains the reason why EpCAM^+^ HCCs are more sensitive to Wnt-beta-catenin signaling antagonists than EpCAM^−^ HCCs [141]. These findings have prompted to isolate EpCAM^+^ cells from EpCAM^+^, Alpha-Fetoprotein^+^ tumors and to define their biological properties: EpCAM^+^ cells displayed properties of CSCs and were able to initiate a tumoral process when inoculated into immunodeficient mice [142,143]. Studies carried out on hepatocarcinoma cell lines suggest that the highest tumor-initiating activity is displayed by CD133^+^EpCAM^+^, compared to CD133^+^EpCAM^−^ and CD133^−^EpCAM^+^ cell populations [144]. According to these findings it was proposed that EpCAM (CD326) could represent an important target in the context of the development of experimental immunotherapy of hepatoblastoma [145].

Other studies have revealed that CD90 is a reliable marker of liver CSCs, and the concomitantly expressed CD44 modulates the biological activity of the CD90^+^ CSCs. CD90 (Thy-1) is a glcosylphosphatidylinositol-anchored glycoprotein expressed by bone marrow-derived mesenchymal stem cells and by normal hepatic stem cells. Given this background, it was logical to investigate the expression of CD90 in hepatocarcinoma cell lines and at the level of primary tumor specimens: enriched populations of CSC-CD90^+^ cells isolated from HCC lines possess tumorigenic activity when transplanted into immunodeficient mice; the large majority of these CD90^+^ cells coexpress CD44 [146]. Interestingly, CD44 regulates the survival and the tumorigenic activity of CD90^+^ liver cancer cells [146]. As it is expected, liver cancer specimens display an increased number of CD90^+^CD45^−^ cells, compared to the normal hepatic tissue [146]. Importantly, in most of hepatocarcinoma patients, circulating CD90^+^CD45^−^ cells were detected [143]. CD90^+^CD44^+^ cells showed a more aggressive phenotype than the CD90^+^CD44^−^ counterpart and developed metastases in the lung of immunodeficient mice [146]. Tumor spheres obtained by the culture of HCC cell lines under serum-free conditions favoring stem cell growth are enriched in CD90^+^ and CD133^+^ cells; Oct4 and ABCG2 were highly overexpressed in CD90^+^ cells and this explains their chemoresistance [147].

Recently, in primary hepatic cancer samples the relative cellular expression of CD90 and EpCAM was analyzed: CD90 positivity does not overlap with EpCAM positivity [148]. Gene expression analysis showed that sorted EpCAM^+^ cells exhibit epithelial features, while CD90^+^ cells display a vascular endothelial pattern of gene expression [148]. Serial xenotransplantation experiments of EpCAM^+^/CD90^+^ cells showed a rapid growth of EpCAM^+^ cells at the level of subcutaneous lesions and a highly metastatic capacity of CD90^+^ cells at the level of the lung [148]. Clinico-pathological analysis showed that the presence of EpCAM^+^ cells was associated with a poorly differentiated phenotype and high serum alpha-fetoprotein levels, while the presence of CD90^+^ cells was associated with a high incidence of distant organ metastases [148]. Cyclin D1 overexpresasion increases the development of the CD90^+^/EpCAM^+^ cell population, increasing stemness and chemoresistance [149]. Cyclin D1 interacts with and activates Smad 2/3 and Smad 4, resulting in the activation of a cell signaling pathway that stimulates liver CSC self-renewal [149]. Smad inhibitors reduced CSC proliferation and increased their chemosensitivity [149].

To identify some genes involved in the carcinogenic process involving CD90 hepatic progenitor, the gene expression profile of tumoral and non-tumoral CD90^+^ cells were compared. About 500 genes were differentially expressed at the level of these two cell populations and, particularly, the genes overexpressed in CD90^+^ CSCs pertain to three main groups, associated with inflammation, drug resistance and lipid metabolism [150].

Other studies have explored other membrane markers of liver CSCs that have led to identify CD13 as a membrane marker for liver CSCs. In an initial study these authors have shown that the side population (SP) in hepatic cancer enriches for CSCs: particularly, cells of the SP fraction express both hepatocyte and cholangiocyte markers, exhibit high resistance to anti-cancer agents, and display high tumorigenicity in xenotransplantation assays in NOD/SCID mice [104]. In a subsequent study these authors identified CD13 as a membrane marker preferentially expressed on SP cells; CD13^++^ cells were quiescent (G_0_/G_1_) SP cells [119]. CD13^+^/CD133^+^ and CD13^−^/CD133^+^ cells were isolated from several hepatocarcinoma cell lines: the former ones grow more slowly in the immunodeficient mice and contained a higher frequency of tumor-initiating cells [119]. The CD13^+^ cells that are CD90^−^ form tumor spheres when plated under non-adherent cell culture conditions: during their growth, in vitro these cells became CD90^+^ [119]. CD13^+^ cells were basically chemoresistant; following treatment, these cells survived and were accumulated along the fibrous capsule, a tissutal region where liver cancers usually relapse [136]. From a functional point of view, CD13 reduced the extent of DNA damage induced by ROS production following genotoxic stress and, through this mechanism, protected cells from apoptosis [119]. In xenotransplantation mouse models, CD13 inhibitors greatly enhanced the anti-tumor effect of 5-Fluorouracil, a drug currently used for treatment of gastrointestinal tumors [151]. Inducers of EMT, such as TGF-beta, stimulate CD13 expression on cancer liver cells [148]. The increased CD13 expression induces an increased protection against apoptosis of liver CSCs. Furthermore, immunohistochemical analysis showed that after chemotherapy treatment CD13 and N-Cadherin were co-expressed at the level of fibrous capsules [147]. These observations suggest a link between EMT and the cancer stem cell marker CD13 in liver cancer [152].

Other studies have led to the identification of OV6 as a marker of liver CSCs. This membrane marker was discovered in the context of studies aiming to define the stimuli inducing the proliferation and differentiation of adult hepatic progenitors (oval cells). It was shown that active Wnt/beta-catenin signaling occurs preferentially at the level of oval cells and, particularly, at the level of a subset of these cells and, particularly, at the level of a subset of these cells that can be labeled by the OV6 monoclonal antibody [153]. OV6^+^ cells have been identified also in the HCC cell lines and in primary liver cancer tumors [138]: these cells have a greater ability to form tumors in vivo and are more chemoresistant that the OV6^−^ counterpart [136]. Wnt/beta-catenin activation is required for the survival and proliferation of these OV6^+^ cells [137]. In a subsequent study the properties of OV6^+^ cells were further characterized, providing evidence that these cells have an invasive and metastatic potential in vivo [153]. Patients with elevated numbers of OV6^+^ cells have tumor with clinico-pathological features of biological aggressivity and have a poor prognosis [153]. Interestingly, SDF-1alpha, the ligand of the chemokine receptor CXCR4, induced the expansion of tumoral OV6^+^ cells and stimulated stemness properties [153].

As for other types of cancer stem cells, also for liver cancer stem cells some evidences have shown that these cells can be identified based on their high ALDH expression. Thus, Ma and coworkers have shown through the analysis of CD133^+^ and CD133^−^ cell populations isolated from Huh7 HCC cells that the former one preferentially expressed ALDH1 [154]. Most of ALDH^+^ cells were also CD133^+^; only a part of CD133^+^ cells were ALDH1^+^ [151]. CD133^+^ ALDH1^+^ cells were more tumorigenic than CD133^+^ ALDH1^−^ cells [155].

Since many markers were reported to identify liver CSCs, some recent studies have addressed the important problem to verify which of these markers is more helpful for this purpose. To this end, the most commonly used markers, including CD133, CD44, EpCAM, CD90, ALDH, have been used to identify by immunohistochemistry CSCs in hepatocarcinomas [156]. None of these markers, when used alone, in various tumors can be considered as a universal marker of CSCs; often reactivity for these markers was observed also in the non-tumoral adjacent areas [156]. This great heterogeneity of the CSC phenotype may reflect a consistent heterogeneity of the tumoral process and indicates that none of these markers can be regarded as a universal marker for liver CSCs [156].

Very recently, it was reported the identification of a new marker of liver CSCs, isolated according to a peculiar experimental strategy. In fact, Zhao and coworkers have isolated from a same patient two cell lines, one from primary tumor cells (Hep 11) and the other one from recurrent tumor cells (Hep12). Then, they have screened a library of MoAbs raised against these two cell lines and by a subtractive immunization approach have identified an antibody, 1B50-1, selectively reacting with recurrent tumor cells [142]. This antibody recognizes the calcium channel α2δ1 subunit and labels a minority of cells in various HCC cell lines; the cells isolated according to their positivity for 1B50-1 display molecular, phenotypic and functional properties of CSCs [157]. Interestingly, in HCC cell lines, 1B50-1^+^ cells represent fractions of CD133^+^1B50-1^+^, CD13^+^1B50-1^+^ and EpCAM^+^1B50-1^+^ cells are the most tumorigenic [157]. Importantly, also 1B50-1^+^ cells isolated from primary liver tumors are tumorigenic in immunodeficient mice [140]. The calcium channel α2δ1 subunit, the antigen recognized by the MoAb 1B50-1 plays a relevant role in the proliferation of liver CSCs, as suggested by the observation that the 1B50-1 MoAb inhibits the growth of tumor spheres [157]. This important biologic effect of α2δ1 is related to the functional role of this calcium channel in the regulation of calcium influx at the level of liver tumor-initiating cells [157]. The functional role of this calcium channel is further supported by the observation that α2δ1 knockdown inhibits ERK phosphorylation in TICs and induces their apoptosis [157].

The biology of liver cancer stem cells remains largely unknown. A recent study used the differential analysis of transcriptome gene expression profiles of liver cancer stem cells and non-cancer stem cell HCC cell lines to identify genes selectively or preferentially expressed in cancer stem cells; using this approach, the transcription factor ZIC2 was found to be highly expressed in liver cancer stem cells [158]. ZIC2 knockdown induced a reduction of liver sphere formation and of tumor xenograft in mice, thus supporting the idea that transcription factor is required for liver cancer stem cell self-renewal [158]. Molecular studies delineated the mechanism of ZIC2 activity on liver cancer stem cells: ZIC2 recruits the nuclear remodeling transcription factor (NURF) complex to the OCT4 promoter, promoting OCT4 activation [158]. OCT4 transcription factor is essential for stemness. In line with these observations, ZIC2, OCT4 and NURF are related to HCC prognosis [158].

Recent studies indicate a key role of the human homologue of the Drosophila spalt homeotic gene SLL4, encoding a CHC2 zinc-finger transcription factor, in the control of liver cancer stem cells. This transcription factor, through its interaction with the transcription factors OCT4, SOX2 and NANOG acts as a regulator of the stemness of embryonic stem cells. At the level of the hepatic tissue, SALL4 is highly expressed in fetal liver and silenced in adult liver, and plays a key role in hepatic cell lineage commitment. However, SALL4 is expressed in a subset of HCC patients and its expression is associated with a negative prognosis: gene expression studies showed that SALL4-positive HCCs display a progenitor-like signature with overexpression of proliferation and metastatic genes [159]. Other recent studies have confirmed the SALL4 expression in a subset of HCCs, characterized by EpCAM positivity [160]. In fact, about 20% of HCCs were shown to be SALL4-positive, while about 16% of these tumors were EpCAM positive [161]. Interestingly, in HCCs, SALL4 expression was positively correlated with EpCAM expression, but not with K19 expression [149]. HCCs expressing both SALL4 and EpCAM displayed a significantly lower survival than tumors negative for both these markers [161]. A recent report showed that SALL4 is frequently expressed in iCCs (58%): SALL4-positive iCC cases display more frequent lymph nodal metastasis and vasculature invasion; furthermore, strongly SLL4-positive iCCs had shorter survival, compared to moderately positive or SALL4-negative cases [162]. Loss of function studies showed a key functional role of SALL4 in sustaining the survival, proliferation and tumorigenicity of HCCs; blocking of SALL4-corepressor interactions released suppression of PTEN expression and elicited an inhibition of tumor formation in xenograft tumor models [159].

As above discussed, fibrolamellar hepatocellular carcinomas (FL-HCCs) are a peculiar form of HCCs, occurring increasingly in children to young adults. A recent report provided initial evidence that these HCCs are particularly enriched in CSCs: particularly, these tumor cells highly express endodermal stem cell markers [163]. Furthermore, transcriptomic analyses of tumor lines isolated from FL-HCCs re-veiled a signature for FL-HCCs closely resembling that of biliary tree stem cells [163].

The biology of liver CSCs is scarcely known and, particularly, the factors involved in the maintenance of self-renewal are poorly characterized. However, several recent reports identified some pathways or some factors playing a critical role in the control of self-renewal of these cells. Thus, some of these studies identified some long noncoding RNAs (lncRNAs) were identified as controller of self-renewal of liver CSCs: lnc-Catm, a lncRNA highly expressed in HCCs, is required for liver CSCs through a mechanism involving its association with β-catenin, the consequent stabilization of this protein, with activation of Wnt-β-catenin signaling [164]. Another lncRNA, lnc RNA Inc TCF7 promotes self-renewal of liver CSCs through activation of Wnt signaling [165]. In addition, some pathways, such as MEK1 [166], NOTCH [167] and SOX9 [168] seem to play a relevant role in the control of self-renewal, maintenance of stemness properties and tumorigenicity of liver CSCs. The aberrant regulation of signaling pathways associated with self-renewal of liver stem cells can drive their aberrant proliferation and even malignant transformation. A highly transcribed lncRNA, lncBRM, associates with BRM and modulates the BRG1/BRM switch in the BRG1-associated factor (BAF) complex, leading activation of YAP1 signaling and promotion of liver CSC self-renewal [169]. Expression levels of Lnc BRM together with YAP1 signaling targets are correlated with tumor severity og HCC patients and, therefore, lnc BRM and YAP1 signaling may serve as biomarkers for diagnosis and potential drug targets for HCC [169].

Recent studies highlight the importance of a metabolic reprogramming of oxidative metabolism in the genesis of hepatic carcinogenesis. Thus, the chronic activation of antioxidant mechanisms could play a key role in the mechanisms of hepatocarcinogenesis [170,171]. In fact, it was shown that transient NRF2 activation (NRF2 is a key positive regulator of cellular antioxidant response) suppresses carcinogenesis via an upregulation of the expression of various antioxidant proteins involved in the neutralization of ROS and in the maintenance of normal levels of reduced glutathione; however, chronic NRF2 activation, elicited by p62 accumulation or by KEAP1 and/or NRF2 mutations, stimulates hepatic carcinogenesis by inducing an inhibition of senescence or death of initially transformed hepatic cells elicited by oxidative stress and allowing these cells to progressively accumulate additional oncogenic mutations required for their oncogenic transformation [172]. Particularly, in the absence of NRF2-induced protection against oxidative stress, ROS-accumulating hepatic progenitors undergo apoptosis and cannot give rise to tumor development [172]. In line with these observations, KEAP1 or NRF2 mutations have been observed in 14% of HCCs.

These observations were confirmed by two additional recent studies. Thus, Shimizu and coworkers reported that phosphorylated p62 was accumulated in HCC (in about 40% of patients) and nuclear localization of NRF2 was frequently observed (63% of HCCs); furthermore, p62 phosphorylation was positively associated with NRF2 activation [171]. Furthermore, in a rat model of hepatocarginogenesis it was shown that early preneoplastic foci and nodules progressing to HCC are characterized by inhibition of oxidative phosphorylation and by enhanced glucose utilization to stimulate the pentose phosphate pathway; these changes are supported by increased expression of the mitochondrial chaperone TRAP1 and of NRF2 that induces the expression of G6PD [172].

The characterization of cells originating liver cancers is essential not only for a better understanding of tumor development, but also for the development of cell platforms capable of predicting the sensitivity of tumor cells to various anti-cancer agents. The development of clinically relevant cellular models for HCC and iCCA is an absolute need for the development of effective treatment strategies. In this context, tumor cell lines do not represent a suitable model their poor predictive power on clinical outcome. To bypass these limitations, in these last years there was the development of patient-derived xenograft models (PDX) as a platform to improve the drug development process [173] and to assist clinical trials [174]. Although PDX models have a good clinical predictivity, their current use to guide clinical trials is considerably limited by the long period of time (4–8 months) required for their establishment. Therefore, a clinically-compatible approach could consist in the development of PDX and of the matching primary cell line [175]. PDX models have been developed for HCC [176] and HB [177,178]. The development of PDX models in HBs was important because allowed the identification of novel therapeutic targets, such as the NRAS mutations [177,178].

In conclusion, the studies carried out in the last years in liver cancers support the theory of cancer stem cells. A number of markers, including CD13, CD44, CD24, CD90, CD133, EpCAM, DLK1, ALDH1 can be used to identify liver cancer stem cells. Although there is no international consensus abouty an ideal marker for liver cancer stem cells, the most promising seem CD90, a marker of oval cells, CD133 and EpCAM. The identification of potential CSC markers offers the opportunity for the development of therapeutic strategies aiming to eradicate these cells from liver tumors. In this context, some recent studies have shown promising approaches based either on nanotechnology or immunologic targeting. Thus, epirubicin-adsorbed nanodiamonds displayed high efficacy in inducing the killing of chemoresistant liver CSCs [179]. Poly lactic-co-glycolic acid-encapsulated disulfiram strongly inhibited liver CSCs and in vivo growth of HCCs [180]. Chimeric antigen receptor (CAR) T cells targeting Glypican 3 (GPC3) suppress HCC growth in vivo [181]. Annexin 3-transfected dendritic cells (Annexin 3 was preferentially expressed on liver CSCs and its level of expression correlated with CD133) induce functionally active T cells able to target liver CSCs [182]. Therefore, CSC membrane markers may a represent CSC-specific therapeutic targets for improving the treatment of liver cancers.

## 6. Conclusions

Both HCC and CCA are genetically and biologically heterogeneous. The marked inter-tumor heterogeneity of HCC and CCA is attributed to the presence of multiple, different etiologies, such as infectious agents, like HBV, HCV and parasitic infections or chemical factors. Furthermore, other risk factors, such as dietary factors, cigarette smoking and excessive alcohol intake, favor the development of these tumors. HCC accounts for 90% of liver cancer in the world, and HBV and HCV are its major causative agents, while CCA is rare, with exception of South-East Asia, where liver fluke infection is endemic and CCA incidence accounts for 70% of all liver cancer cases. Importantly, the presence of causative agents, such as parasitic infection, affects the genetic alterations observed in CCAs.

In the last two decades, there have been enormous progresses in our understanding of the cellular and molecular basis of liver cancers. NGS-based studies have provided evidence on the genes that are more frequently altered in the various liver cancer, providing evidence about the complexity and the specificity of these alterations. These studies have shown that in consequence of these genetic and epigenetic alterations, some signaling pathways are activated and their activation plays a functional role in hepatocarcinogenesis, as supported by functional studies. The combination of cellular and molecular studies led to the development of in vivo lineage tracing experiments that have in part clarified the cellular origin of liver cancers, showing an unexpected plasticity of mature hepatocytes. In addition to mature hepatocytes, also hepatic progenitors may be involved in the genesis of various liver cancers.

The molecular studies led to the identification of three subsets of HCCs and two subsets of CCAs that can be distinguished according to genetic alterations, gene expression and methylation profiles to categories such as: (i) proliferation-progenitor, proliferation-transforming growth factor β and Wnt-catenin β1 for HCC; (ii) proliferation and inflammation for CCA. Furthermore, integrated genomic studies have led to the identification in HCCs of potential therapeutic targets for which inhibitors exist, including WNT signaling, MDM4, MET, VEGFA, MCL1, TERT and immune check proteins CTLA-4, PD-1 and PD-L1. Potential therapeutic targets for iCCAs are IDH1 and IDH2, MET, FGFR2, VEGF and its receptors and MEK 1 and MEK2.

In a future perspective, an integrated omics approach, combining and correlating genomics, epigenomics, transcriptomics, proteomics and metabolomics data, will provide a key to better define tumor heterogeneity and to identify cancer drivers and to define optimized individualized multitarget therapies for HCC and iCCA patients.

The definition of tumor heterogeneity in each patient is of fundamental importance because it will allow to define trunk mutations and branch mutations: trunk mutations promote tumor progression and should be the first targets of molecular therapies. Furthermore, it will be important to perform studies with a patient enrichment strategy, based on biomarkers that predict the response. This conclusion is supported by the observation that the two systemic therapies the improve survival in patients with HCC in first-line (sorafenib) and second-line (regorafenib − Stivarga®) regimens have been tested in patient populations enrolled according to any selection strategy [183,184]. Interestingly, meta-analysis of two studies analyzing the effect of sorafenib on the OS of HCC patients with unresectable tumors showed that significantly greater OS sorafenib benefit versus placebo was observed in patients without extrahepatic spread, with hepatitis C and with a lower neutrophil to lymphocyte ratio [185].

It is interesting to note that the importance of molecular studies is fundamental not only for the definition of the genetic alterations associated with liver cancer development, but also for the definition of the risk of developing HCC in the context of cirrhotic liver. In fact, a recent study, based on the transcriptomic meta-analysis of >500 human cirrhotic livers, showed the existence of regulatory gene modules driving HCC risk and the existence of the lysophosphatidic acid pathway as a central chemoprevention target [186].
